# Strain improvement of *Escherichia coli* K-12 for recombinant production of deuterated proteins

**DOI:** 10.1038/s41598-019-54196-w

**Published:** 2019-11-27

**Authors:** Vinardas Kelpšas, Claes von Wachenfeldt

**Affiliations:** The Microbiology Group, Department of Biology, Lund University, Sölvegatan 35, SE-223 62 Lund, Sweden

**Keywords:** Structural biology, Expression systems, Applied microbiology

## Abstract

Deuterium isotope labelling is important for structural biology methods such as neutron protein crystallography, nuclear magnetic resonance and small angle neutron scattering studies of proteins. Deuterium is a natural low abundance stable hydrogen isotope that in high concentrations negatively affect growth of cells. The generation time for *Escherichia coli* K-12 in deuterated medium is substantially increased compared to cells grown in hydrogenated (protiated) medium. By using a mutagenesis plasmid based approach we have isolated an *E. coli* strain derived from *E. coli* K-12 substrain MG1655 that show increased fitness in deuterium based growth media, without general adaptation to media components. By whole-genome sequencing we identified the genomic changes in the obtained strain and show that it can be used for recombinant production of perdeuterated proteins in amounts typically needed for structural biology studies.

## Introduction

Structural biology techniques such as neutron macromolecular crystallography (NMX), nuclear magnetic resonance (NMR) spectroscopy and small angle neutron scattering benefit from isotopic substitution of hydrogen atoms within a macromolecule for deuterium atoms. Deuterium substitution can be used to simplify NMR spectra owing to that deuterium NMR absorptions are not detected under the conditions used for proton NMR. Due to the low scattering power of hydrogen for X-rays, hydrogens typically are not seen in protein structures determined by X-ray crystallography except at subatomic resolution^1^. NMX can be used to locate hydrogen atoms accurately in proteins or other molecules^2^. However, NMX is limited by that hydrogen (protium or ^1^H that is the most prevalent hydrogen isotope is here referred to as hydrogen) in protein or associated water molecules causes high background due to the large incoherent scattering of neutrons. In addition, the coherent scattering length of hydrogen is negative reducing the effective scattering power of the crystal^3^. These problems can be overcome by exchanging hydrogen for deuterium^2,3^. Labile hydrogen atoms (backbone amides, OH-, NH-, and SH-groups of polar amino acid side chains) in proteins can generally be substituted by replacing the water solvent with D_2_O (heavy water). However, the labile H atoms typically account for a minority of the total hydrogens in a protein. To replace all (>99%) hydrogen atoms by deuterium (perdeuteration) recombinant protein production is done using deuterated growth media. Typically, cells are grown in a defined D_2_O-based minimal medium supplemented with a perdeuterated carbon source. Except for yeast and single cell algae, most eukaryotic cells do not grow at deuterium levels high enough to be of any practical use in downstream applications. *Escherichia coli*, the most commonly used host for recombinant protein production, can be grown in a fully deuterated minimal medium. However, growth rate slows down markedly in deuterated media which can be attributed to the altered properties of D_2_O as solvent and the replacement of all hydrogens (^1^H) in other molecules with deuterium (^2^H)^4^. Several studies have reported on the biological effects of deuterium on microorganisms but the underlying mechanisms of growth reduction remain poorly understood. In yeast which was adapted to growth in deuterated media an increased production of the heat shock protein, Hsp70 was observed^5^. In bacteria it was reported that deuterium in the growth medium increases catalase activity and induce effects similar to that of osmotic shock^6^.

A common strategy for obtaining deuterium labelled proteins involves adaptation of the expression host to growth in deuterated medium by step-wise increasing the proportion of D_2_O in the growth medium. This is a time-consuming process that has to be repeated for each protein to be produced^7^. Long term adaptation of *E. coli* for growth in deuterated media has been reported^8^. However, the genetic basis for adaptation was not investigated.

The objective of this work was to obtain an *E. coli* strain or collection of strains with improved growth performance in deuterated media. Rational design of strains or genes for specific purposes requires considerable knowledge about the relevant physiological, metabolic and genetic components. When there is limited knowledge or a rational design approach does not provide further improvements, random mutagenesis can be successfully employed^9^. Here we performed random mutagenesis on the *E. coli* K-12 strain MG1655 to find mutations supporting improved growth in minimal D_2_O-based media. The *E. coli* K-12 strain MG1655 was used in this study because its genome has been completely sequenced and is very well annotated^10^. A plasmid-based mutagenesis approach was used to obtain deuterium tolerant strains. The mutator plasmid encodes five genes that affect, base excision repair, base selection, and mismatch repair, and proofreading capabilities thereby elevating the mutation rate^11^. We found that strains with clearly enhanced growth properties in minimal D_2_O-based media could be obtained. One of the strains was studied in detail and modified for T7 RNA polymerase directed recombinant gene expression. We show that the resulting strain, *E. coli* DA1 (Deuterium Adapted strain 1), grows faster compared to the parental *E. coli* MG1655 strain, and that it can be used for efficient production of recombinant perdeuterated proteins.

## Materials and Methods

### Bacterial strains, culture conditions and transformation

Media were supplemented when needed with the appropriate antibiotic at the following concentrations: 50 µg/ml kanamycin, 40 µg/ml chloramphenicol. Lysogeny broth (LB), NaCl 10 g/l, Difco yeast extract 5 g/l, Difco tryptone 10 g/l, made up with H_2_O, pH 7.6, was used as a rich medium. The defined M9 minimal medium (referred to as H-M9) consisted of 42.7 mM Na_2_HPO_4_ • 2H_2_O, 22 mM KH_2_PO_4_, 8.6 mM NaCl, 107 mM NH_4_Cl, 1 mM MgSO_4_ • 7H_2_O, 0.1 mM CaCl_2_, 2 mg/l thiamine HCl, 0.018 mM FeCl_3_ • 6H_2_O, and glycerol 0.8% (w/v) as sole carbon source. To induce expression of recombinant protein 0.5 mM isopropyl β-D-1-thiogalactopyranoside (IPTG) was added to the growth media. When deuterated media were prepared all solutions were made in heavy water (99.8% D-atom, Sigma-Aldrich) and then filtered with 0.22 µm sterile filter (VWR Vacuum filtration unit). In the deuterated media either regular glycerol (medium referred to as D-M9) or glycerol d-8 (98% D-atom, Sigma-Aldrich) (medium referred to as DD-M9) was used. All salts used in D-M9 and DD-M9 were the same as used for M9, but instead of Na_2_HPO_4_ • 2H_2_O, anhydrous Na_2_HPO_4_ was used. For perdeuterated protein production a 1 M stock solution of IPTG was prepared in heavy water. Plates and liquid cultures were incubated at 30 °C or 37 °C. Strains were revived from glycerol stocks by streaking onto Lysogeny broth agar (LA) and incubation at 37 °C. Plasmid DNA was transformed into electrocompetent cells which were prepared essentially as described by Warren^12^.

### Preparation of D-M9 agar

Solutions with deuterium were not autoclaved to avoid isotope exchange with hydrogen molecules from water. All solutions used to prepare D-M9 agar plates were filtered through a 0.2 µm pore size filter. Bacto® Agar (Difco) was irradiated with UV (254 nm) three times for five minutes using a CL-508 Cross linker (Uvitec, Cambridge). A solution of agar in heavy water was heated at 85 °C until the agar was melted and then mixed together with media solutions brought to 50 °C and poured in Petri dishes.

### Growth experiments

Bacterial cultures were started by inoculating a few colonies from a LA plate of the appropriate strain using a sterile pipette tip into 25 ml H-M9. The cultures were incubated at 37 °C in an incubator shaker (Infors, Multitron Standard) at 200 rpm for 12 to 18 hr. After incubation the cultures were diluted approximately 30 times to an optical density at 600 nm (OD_600_) of 0.1 in fresh H-M9 medium and incubated at 37 °C with shaking (200 rpm) and OD_600_ versus time was recorded. When growth was performed in deuterated media the following steps were carried out to minimize carry-over of hydrogen. The initial cultures grown in H-M9 medium were harvested by centrifugation (8 min at 8000 × g at 20 °C), the supernatant was carefully removed and the cell pellet was suspended in 10 ml D-M9 medium. The cultures were incubated at 37 °C with shaking (200 rpm), until they reached an OD_600_ between 1 and 3. These cells which were in the exponential growth phase were diluted to an OD_600_ of 0.1 in a total volume of 25 ml DD-M9 medium and incubated at 37 °C, 200 rpm. Cultures of 25 ml total volume were cultivated in 250 ml baffled Erlenmeyer flasks (Bellco Glass Inc.) and 10 ml cultures were grown in 100 ml Erlenmeyer flasks (Schott DURAN). The flasks were incubated at 37 °C in an incubator shaker at 200 rpm. Growth rate experiments were performed by measuring the OD_600_ of duplicate or triplicate cultures over several time points at cell densities between 0.05 and 2. The specific growth rate (*μ*) was calculated as the slope of the linear best-fit line through a plot of ln (OD_600_) versus time (hours). The generation time (or doubling), *t*_d_, is equal to ln2/*μ⊡* Growth curves are shown as semilogarithmic plots of time versus OD.

### Random mutagenesis

To induce random mutations in chromosomal DNA, the parental strain *E. coli* MG1655, was transformed with the inducible mutagenesis plasmid MP6^11^. *E. coli* MG1655/MP6 cultures were supplemented with 40 µg/ml of chloramphenicol. The mutagenesis plasmid system was tested in terms of efficiency in acquiring mutants resistant to rifampicin. *E. coli* MG1655 (control for spontaneous mutations) and *E. coli* MG1655/MP6 were grown in H-M9 at 30 °C, 200 rpm. 25 ml cultures were started at an OD_600_ of 0.1. At an OD_600_ of 0.5 arabinose to a concentration of 100 mM was added to induce expression of the plasmid encoded dominant mutator alleles. 18 hours post induction serial dilutions were made and plated on LA and LA + 100 µg/ml rifampicin. Single colonies were counted (averages were estimated from two plates with a dilution factor difference of 10). The viable count and the number of rifampicin resistant colonies (Rif^R^) was used to estimate the mutation rate. For mutagenesis in deuterium based growth medium *E. coli* MG1655/MP6 was grown in 25 ml DD-M9, supplemented with 40 µg/ml of chloramphenicol, at 30 °C until OD_600_ reached 0.5. At this point arabinose to a final concentration of 100 mM was added. After 20 hours, serial dilutions were done and cells were plated on D-M9 agar plates. Obtained single colonies were picked and streaked on LA plates and used to screen for improved growth using the automated microbiology growth curve analysis system Bioscreen C (Oy Growth Curves Ab Ltd). Each well contained 250 µl DD-M9. Clones which showed increased growth rate were selected for further growth experiments in 250 ml baffled Erlenmeyer flasks. Before making glycerol stocks for long-term freezer storage, strains were tested for chloramphenicol sensitivity, by streaking on LA plates containing 40 µg/ml of chloramphenicol. All cultures during mutagenesis and subsequent analysis were incubated at 30 °C, 200 rpm.

### DNA sequencing

Illumina sequencing technology was used for whole genome shot-gun resequencing and Sanger sequencing was used to confirm mutations in individual genes. Chromosomal RNA-free DNA was purified using the DNeasy Blood and Tissue Kit (Qiagen) according to the manufacturer’s instructions. Illumina sequencing was done at GATC Biotech AG (Germany). Chromosomal DNA isolated from the parental MG1655 strain and the mutagenized strains were sequenced at the Department of Biology, University of Copenhagen (Denmark). The genome sequencer Illumina HiSeq was used with approximately 10 million 2 × 150 bp paired end read output. Reads were mapped using the CLC genomics workbench (Qiagen) to the reference genome sequence of *E. coli* MG1655 (Genbank entry code: U00096.3) obtained from the NCBI genome repository. Observed differences were compared to the parental strain from our laboratory stocked MG1655 strain. In order to confirm mutations particular stretches of DNA were amplified by PCR and then subjected to Sanger sequencing (Eurofins).

### Protein production

The DE3 lysogens of *E. coli* MG1655 and *E. coli* DA1 was prepared using the λDE3-lysogenization kit (Merck). MG1655 and DA1 were grown in LB supplemented with 0.2% maltose, 10 mM MgSO_4_ at 37 °C to an OD_600_ of 0.5. 10 μl of the respective bacterial strain was added to 490 μl of a mixture of phage lysates of λDE3 (6 × 10^10^plaque-forming units (pfu)), Helper phage (10^11^pfu), and Selection phage (10^14^pfu) in 20 mM Tris-HCl, pH 7.4, 100 mM NaCl, 10 mM MgSO_4_. The bacterial cell/phage mixture was incubated at 37 °C for 20 min. 10 μl of each solution was plated onto LA plates. The plates were incubated at 37 °C overnight. A few colonies were transferred to fresh LA plates and the T7 tester phage was used to verify successful lysogenization. The λDE3 cannot be excised from the chromosome by itself and is thus stably maintained in the lysogenized strains. The obtained lysogens *E. coli* MG1655(DE3) and *E. coli* DA1(DE3) were transformed with plasmids pNIC28_Lm_TIM and pETM14_sfGFP. Plasmid pNIC28_Lm_TIM has been described before^13^. Plasmid pETM14_sfGFP was constructed by inserting a fragment encoding sfGFP into plasmid pETM14-ccdB^14^ using sequence and ligation independent cloning^14^. The fragment containing the gene encoding sfGFP was amplified by PCR using primers sfGFP_slic_for.

(5′-CCAGGAACAAACCGGTGGAATGACAAACTATCAGCATGAGCTATACTTCG-3′) and sfGFP_slic_rev.

(5′-CCCCAGAACATCAGGTTAATGGCGTTATCATTTGTACAGTTCATCCATACCATGCGT-3′) and plasmid pCW101_*sfgfp*^15^ as template.

Cultures were inoculated and set-up as outlined under “growth experiments” in DD-M9 medium. At an OD_600_ of 0.5 or at an OD_600_ of 1.2 IPTG to a final concentration of 0.5 mM was added to the cultures. At different time points 1 ml of culture was collected, centrifuged at 8000 × g for 8 min at 4 °C. The supernatant was carefully removed and the cell pellet was suspended in 0.25 ml cold 50 mM Tris-HCl, 100 mM NaCl, 10 mM EDTA buffer (pH 8.0) and stored at −20 °C until used. Samples were thawed on ice, and then sonicated using a Vibra Cell disruptor. Samples were then centrifuged at 20000 × g for 45 min at 4 °C. 40 µl of the supernatant was mixed with 10 µl 5X SDS-PAGE sample buffer (60 mM Tris-HCl (pH 6.8), 25% glycerol, 2% SDS, 0.1% bromophenol blue, 0.7 M 2-mercaptoethanol) and incubated at 95 °C for 10 min. 6 µl sample was loaded per lane on a SDS-PAGE gel (Any kD™ Criterion™ TGX™). Tris-Glycine-SDS was used as running buffer. Bands were visualized by using Coomassie G-250 (Bio-Safe™ Coomassie, Bio-Rad) according to the manufacturer’s protocol. Measurement of sfGFP fluorescence on whole cells was done by suspending cells in 0.5 ml 50 mM Tris-HCl, 100 mM NaCl, 10 mM EDTA buffer (pH 8.0). Samples were then diluted 100-fold in 100 mM NaCl, 100 mM sodium-phosphate buffer (pH 7.5) and OD_600_ as well as sfGFP fluorescence (excitation at 485 nm, emission at 510 nm) were measured. Fluorescence was measured using a RF-5301 Spectrofluorophotometer (Shimadzu) and fluorescence intensity was recorded after subtraction of background fluorescence for the buffer used.

## Results and Discussion

### *In vivo* random mutagenesis and selection for improved growth in deuterated media

**Figure 1:**
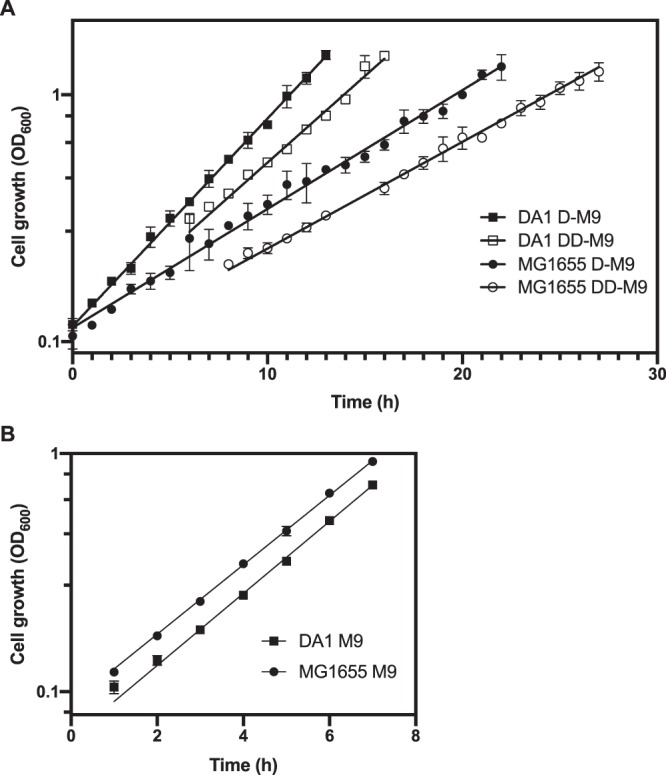
Growth curves of *E. coli* strains. (**A**) *E. coli* MG1655 and *E. coli* DA1 were grown in deuterated minimal medium supplemented with hydrogenated (D-M9) (closed symbols) or deuterated (DD-M9) (open symbols) glycerol. Circles represents the reference strain MG1655 and squares the DA1 strain. The specific growth rates are: 0.194 ± 0.008 h^−1^ (DA1 D-M9), 0.161 ± 0.007 h^−1^ (DA1 DD-M9), 0.111 ± 0.005 h^−1^ (MG1655 D-M9) and 0.099 ± 0.004 h^−1^ (MG1655 DD-M9). Doubling times: 3.58 ± 0.14 h (DA1 D-M9), 4.29 ± 0.18 h (DA1 DD-M9), 6.25 ± 0.26 h (MG1655 D-M9) and 6.96 ± 0.25 (MG1655 DD-M9). (**B**) Cells were grown in minimal medium supplemented with hydrogenated glycerol (M9). Circles represents the reference strain MG1655 and squares the DA1 strain. The specific growth rates are: 0.348 ± 0.011 h^−1^ (DA1) and 0.335 ± 0.012 h^−1^ (MG1655). Doubling times: 1.99 ± 0.06 h (DA1) and 2.06 ± 0.07 h (MG1655). For each strain and medium three biological replicates were analyzed. The mean is plotted and the error bars represents the standard deviation. Error bars are not shown when they are shorter than the size of the symbol. The lines represent the fit to exponential growth. The R-squared values for all fits except for MG1655 grown in D-M9 are ≥ 0.99. For MG1655 grown in D-M9 it is 0.98.

The natural mutation rate in *E. coli* is generally low, approximately 10^−9^ to 10^−10^ substitutions/nucleotide/generation^16^. There are different strategies to increase mutation rates which could shorten the time required to generate mutations needed for improved growth. We used the recently described mutagenesis plasmid MP6 which encodes arabinose inducible dominant mutator alleles that elevate the mutation rate above the natural level^11^. The mutagenesis plasmid was transformed into *E. coli* MG1655 and the mutation rate of the transformed strain was assessed by rifampin resistance screening. The presence of the MP6 plasmid and the inducer arabinose increased the mutation rate three orders of magnitude over the basal mutation rate (Table 1). Next, *E. coli* MG1655/MP6 was grown for 18 hours in minimal deuterium based medium with perdeuterated glycerol as carbon source (DD-M9) in the presence of inducer. The cells were then plated on D-M9 agar plates. The first colonies that appeared after incubating the plates for two days were selected and screened for loss of the MP6 plasmid and altered growth properties in DD-M9. After analyzing 48 clones for growth in DD-M9 using a Bioscreen C automated microbiology growth curve analysis system we found one clone (named DA1) with improved growth properties. In DD-M9, strain DA1 show a ~60% increased specific growth rate (0.161 ± 0.007 h^−1^) compared to the parental strain (0.099 ± 0.004 h^−1^) (Fig. 1A). Growth improvement was also evident in deuterium based medium with un-labelled glycerol (D-M9) (Fig. 1A) with specific growth rate of 0.194 ± 0.008 h^−1^ for strain DA1 compared to 0.111 ± 0.005 h^−1^ for the parental strain. Remarkable, when regular minimal medium (H-M9) was used the growth rate of strain DA1 (0.348 ± 0.011 h^−1^) was similar to that of the parental MG1655 strain (0.335 ± 0.012 h^−1^) (Fig. 1B). Thus, strain DA1 adapted to the growth-restricting effects of deuterium not to the minimal glycerol medium per se. Growth for several generations in the absence of deuterium in either rich broth medium or minimal medium did not affect later growth in deuterated media suggesting that strain DA1 stably maintained growth enhancing mutations.

**Table 1:** Spontaneus and induced mutation rates of *E. coli* strains assesed by rifampicin resistance selection.

Strain	Plate count (CFU ml^−1^) − rifampicin	Plate count (CFU ml^−1^)+ rifampicin	Rif^r^ mutants per plate count	Fold increase
MG1655	2.3 × 10^11^	2100	9.1 × 10^−9^	1
MG1655/MP6	3.1 × 10^9^	37500	1.2 × 10^−5^	1324

### Adaptation on the genomic level

To identify genetic changes in strain DA1 chromosomal DNA was extracted and subjected to whole genome sequencing. As references the genome of the parental MG1655 strain and of a clone (Mut25) derived from MG1655/MP6 that did not show improved growth (Supplementary Fig. 1) were sequenced. The genetic differences between the parental and the two MG1655/MP6 derived strains was established (Table 2, Supplementary Tables 1 and 2). Strain DA1 had fewer mutations as compared to the Mut25 strain and none of the mutations were common to both strains (Table 2, Supplementary Table 2). In strain DA1 we detected two mutations in the intergenic region *edd*/*zwf* and *yehK/yehL* and point mutations in three genes; one nonsense mutation and two missense-mutations. Mutations were present in the following genes; *phoA* (encoding alkaline phosphatase), *metG* (encoding methionine-tRNA ligase) and *rraA* (encoding regulator of ribonuclease activity). The mutations in the *phoA* and *metG* genes are predicted to result in amino acid substitutions while the mutation in the *rraA* gene generates a premature stop codon (Table 2).

**Table 2:** Mutations identified in *E. coli* DA1^a^.

Position^b^	Variant^c^	Reference base(s)	Variant base(s)	Annotations	Coding region change	Protein change^d^
401835	SNV	A	T	*phoA*	89 A > T	Glu30Val
1934671	SNV	G	A	*edd/zwf*	intergenic	
2195414	SNV	A	G	*metG*	1115 A > G	Asp372Gly
2204449	SNV	T	C	*yehK/yehL*	intergenic	
4118955	SNV	C	A	*rraA*	376 G > T	Glu126*

^a^In addition to the listed mutations, the *E. coli* DA1 strain had the same changes relative to the reference genome sequence of *E. coli* MG1655 (Genbank entry: U00096.3) as was identified in the used laboratory stock of MG1655 (Supplemental Table 1).

^b^Nucleotide position in the reference sequence Genbank entry: U00096.3.

^c^Single nucleotide variant (SNV).

^d^Non-sense mutation given a truncated polypeptide (*).

Alkaline phosphatase (PhoA) is a dimeric periplasmic protein that is important in the response to inorganic phosphate starvation^17^. The mature PhoA without the signal peptide starts at Arg22. Glu30 is placed far from both the active site and the dimerization face. The mutation in *phoA* resulting in the exchange of Glu30 for Val is likely to have little effect on PhoA. A plate assay to detect alkaline phosphates activity using the indicator dye 5-bromo-4-chloro-3-indolylphosphate-p-toluidine did not show any difference between the wild-type and the DA1 strain. Thus, we find it unlikely that the mutation in *phoA* is critical for the observed growth improvement of strain DA1.

**Figure 2:**
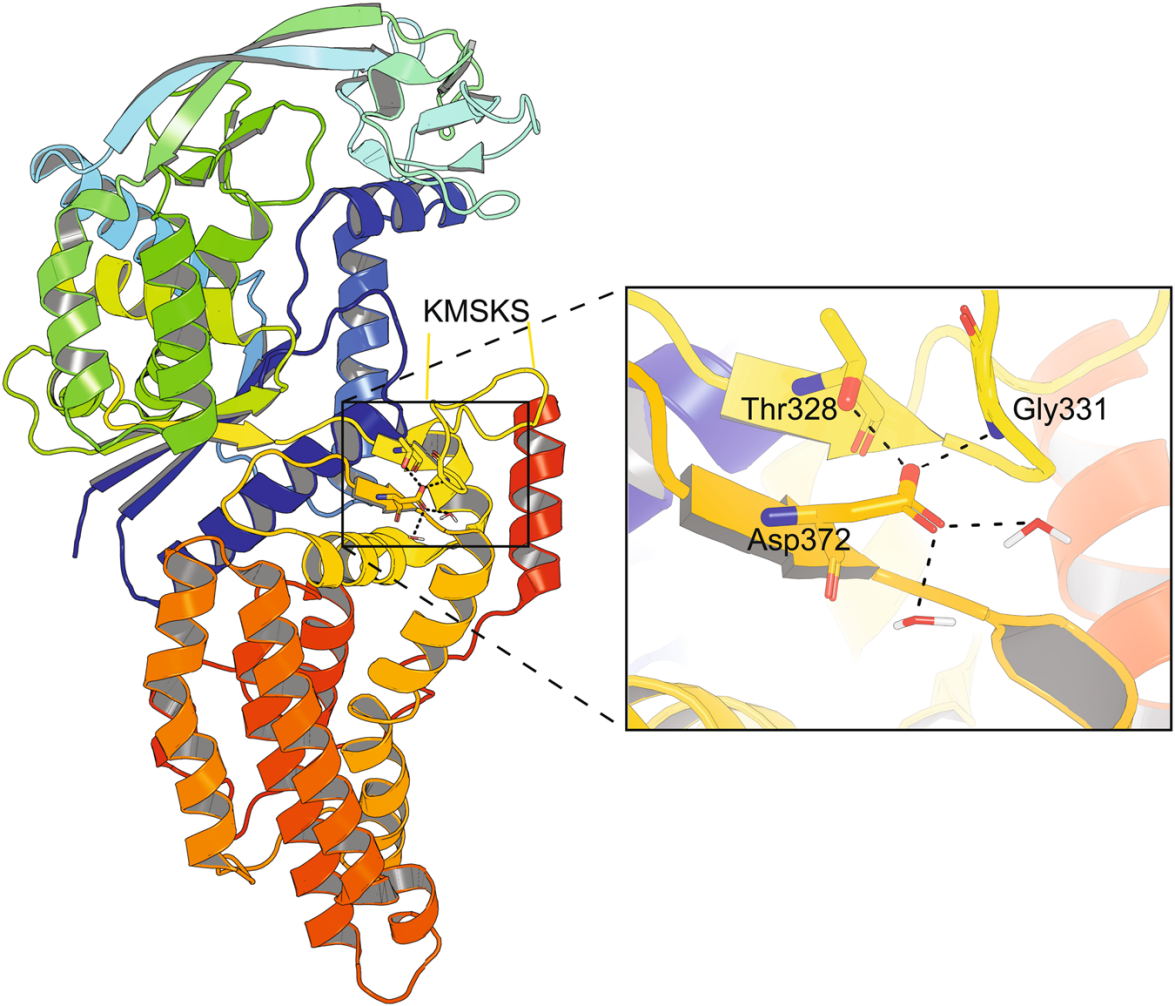
*E. coli* MetRS crystal structure highlighting Asp372. Rainbow colored cartoon representation from blue (N-terminus) to red (C-terminus) of *E. coli* methionyl-tRNA synthetase (PDB entry 1F4L). The catalytic part of MetRS is shown in the upper part of the figure (green – blue) and the anticodon domain is shown in the lower part of the figure (orange). The position of the KMSKS loop is indicated. Inset shows the putative hydrogen bonding network (dashed black lines) involving the side chain of Asp372.

The *metG* gene encodes the essential methionyl-tRNA synthetase (MetRS) that has a key role in the translation initiation process^18^. The enzyme is comprised of a catalytic Rossmann fold domain and a C-terminal α-helix bundle domain, responsible for the recognition of the CAU anticodon of methionine tRNAs^19^. The two domains are connected by a β–α–α–β–α topology (SC fold) domain that contains the evolutionarily conserved KMSKS motif that is important for the catalytic reaction^19,20^. The mutation in *metG* results in an exchange of Asp372 for glycine. The replaced amino acid is located in the β8-strand of the SC fold that play a role in tRNA binding and the aminoacylation reaction (Fig. 2). In the crystal structure of the enzyme (PDB entry 1F4L) the Asp372 side chain is involved in a hydrogen bond network between Thr328 and Gly331 as well as with two water molecules (Fig. 2). The exchange for glycine will disrupt these interactions and is likely to affect the stability of the β-sheet and may enhance the flexibility of the KMSKS loop of the SC fold domain affecting catalysis of methionyl adenylate formation. We hypothesize that reduced dynamics of deuterated methionyl-tRNA synthetase is compensated by the exchange of Asp372 for glycine and that this adaptation contributes to the reduced generation time.

The regulatory protein RraA influences the activity of the essential ribonuclease, RNase E which has a global effect on RNA abundance in *E. coli*. Deletion of *rraA* in *E. coli* results in changes of approximately 80 transcripts that includes the *metG* gene^21^. The nonsense mutation in *rraA* most likely lead to a loss of function and suggests that DA1 has an elevated expression of the mutated *metG*. Further experiments are needed for the identification of the causative role of the identified mutations improving the growth. It is not unlikely that the identified mutations have a synergistic effect underlying the improved growth.

### DA1 a strain suitable for protein perdeuteration

**Figure 3:**
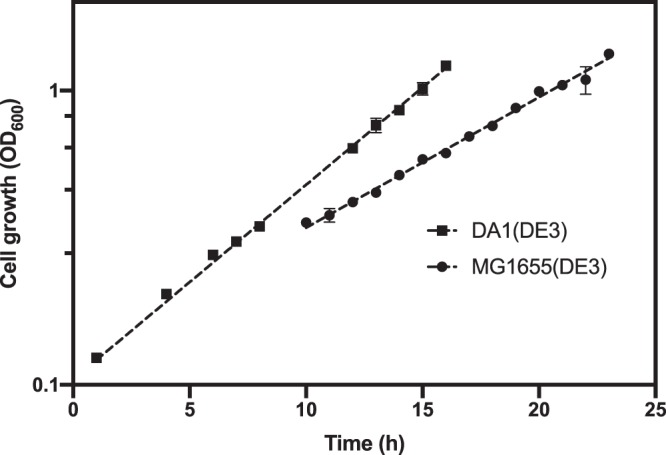
Growth curves of the *E. coli* DE3 lysogens. *E. coli* MG1655(DE3) (circles) and DA1(DE3) (squares) were grown in deuterated minimal medium supplemented with deuterated glycerol (DD-M9). For each strain two biological replicates were analyzed. The mean is plotted and the error bars represents the standard deviation. Error bars are not shown when they are shorter than the size of the symbol. The lines represent the fit to exponential growth. The R-squared values for the fits are 0.99 for DA1(DE3) and 0.98 for MG1655(DE3). The specific growth rates are: 0.152 ± 0.007 h^−1^ for DA1(DE3) and 0.102 ± 0.006 h^−1^ for MG1655(DE3) corresponding to doubling times 4.55 ± 0.21 h for DA1(DE3) and 6.79 ± 0.38 h for MG1655(DE3).

**Figure 4:**
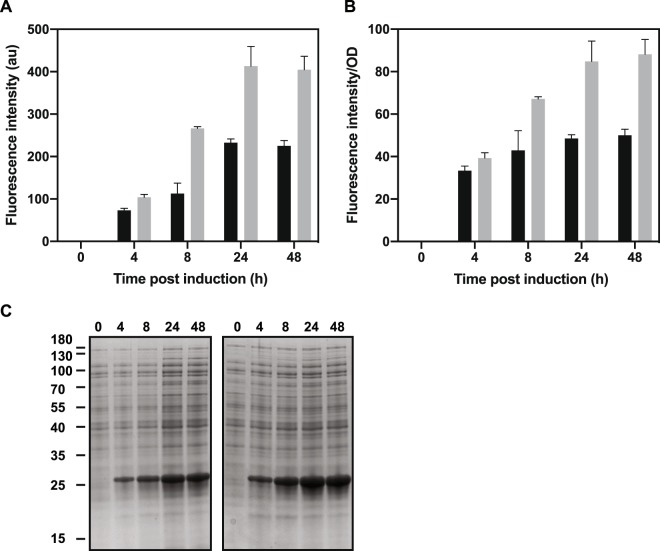
Recombinant protein production in *E. coli* MG1655(DE3) and DA1(DE3). (**A**) Absolute fluorescence intensities (au, arbitrary units) in cells harvested at different time points after inducing expression of the gene encoding sfGFP. (**B**) Fluorescence intensities normalized to cell densities (OD_600_). Three replicates were analyzed and the mean fluorescence intensity is plotted. The error bars represents the standard deviation. MG1655(DE3) black bars. DA1(DE3) grey bars. (**C**) SDS–PAGE analysis of the soluble fraction of lysed cells (MG1655(DE3) left gel, DA1(DE3) right gel) at the indicated time points after induction with IPTG. Each lane was loaded with a sample corresponding to 19 µl cell culture. The positions of molecular mass standards, in kilodaltons, are indicated on the left. The figure was edited for clarity. The unedited figure is shown in Supplemental Fig. 2.

**Figure 5:**
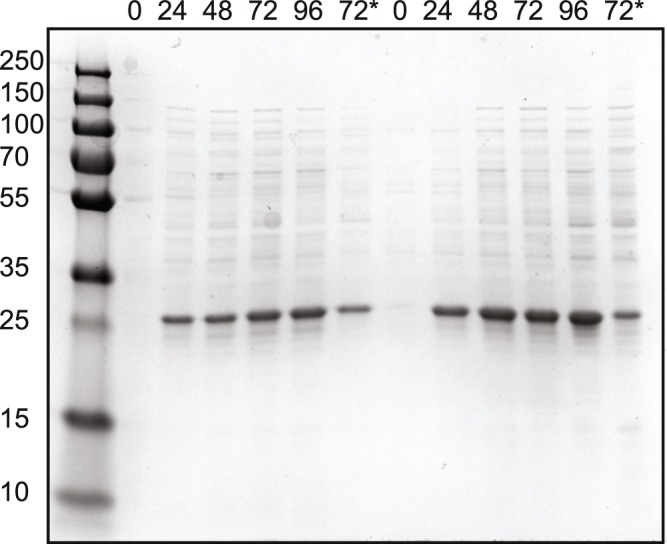
Production of TIM in MG1655(DE3) and DA1(DE3). SDS–PAGE analysis of the soluble fraction of lysed cells at the indicated time points after induction with IPTG. The expression of the gene encoding TIM was induced when OD_600_ was 1.2 except for the 72 hr samples (indicated with a star) that were taken from cells induced at an OD_600_ of 0.5. Each lane was loaded with a sample corresponding to 10 µl cell culture. Soluble fraction from lysed cells of MG1655(DE3) is shown in the left part of the gel and from DA1(DE3) in the right part of the gel. The molecular mass standards, in kilodaltons, are indicated on the left. The uncropped image of the SDS-PAGE gel is shown in Supplemental Fig. 3.

It is a common practice to use T7 RNA polymerase to direct expression of recombinant genes for protein production. Thus, strain DA1 was lysogenized with the λ bacteriophage DE3 that carries the T7 gene 1 encoding the T7 RNA polymerase under control of the IPTG inducible *lac*UV5 promoter^22^. The growth properties of the DE3 lysogens, DA1(DE3) and MG1655(DE3), were similar to the respective non-lysogenic strain (Fig. 3). Next, we investigated if the improved strain was suitable for recombinant protein production in a deuterium based growth medium (DD-M9). Two proteins were used as test cases; superfolder Green fluorescent protein (sfGFP) and Triose-phosphate isomerase (TIM) from *Leishmania mexicana*. The former was chosen because of its ease of detection and TIM because neutron protein crystallography will provide critical understanding of the chemistry of the enzyme-catalyzed reaction. The respective gene for sfGFP and TIM were cloned in low copy number pET like plasmids under the T7 promoter controlled by the *lac* repressor and transformed into DA1(DE3) and MG1655(DE3). Both strains producing sfGFP were grown in DD-M9 at 37 °C and sfGFP levels were analyzed 4, 8, 24 and 48 hrs post induction by SDS-PAGE analysis and by measuring fluorescence of sfGFP. Strain DA1(DE3) produced higher levels of sfGFP and in a shorter time compared to MG1655(DE3) (Fig. 4A). Also when the fluorescence intensity was normalized to the optical density of the cultures strain DA1(DE3) showed higher sfGFP levels (Fig. 4B). The sfGFP levels in intact cells corresponded to the estimated levels of GFP in the soluble extract analyzed by SDS-PAGE (Fig. 4C). Next, the glycolytic enzyme TIM was produced in the two strains grown in DD-M9 at 37 °C. Both strains produced TIM to similar levels. However, the maximum amount of TIM was reached 48 hours post induction in DA1(DE3) compared to 72 hours for MG1655(DE3) (Fig. 5). The total amount of produced protein was similar for the two strains. TIM production negatively affected growth of both strains. Thus, a higher level of TIM was achieved by inducing cells at an OD_600_ of 1.2. Inducing protein production at an OD_600_ of 0.5 resulted in much lower protein yield most likely due to inhibition of growth by the recombinant TIM enzyme. By using strain DA1 perdeuterated TIM has recently been produced in quantities need for neutron crystallography^13^.

In conclusion, we show that *E. coli* DA1 genetically adapted specifically to deuterated growth media. *E. coli* DA1(DE3) has a clearly improved growth rate without compromising recombinant protein production yield in deuterated growth media.

## Supplementary information

Supplementary information

## References

[1] Hirano Y, Takeda K, Miki K (2016). Charge-density analysis of an iron-sulfur protein at an ultra-high resolution of 0.48 A. Nature.

[2] O’Dell WB, Bodenheimer AM, Meilleur F (2016). Neutron protein crystallography: A complementary tool for locating hydrogens in proteins. Arch Biochem Biophys.

[3] Shu F, Ramakrishnan V, Schoenborn BP (2000). Enhanced visibility of hydrogen atoms by neutron crystallography on fully deuterated myoglobin. Proc Natl Acad Sci.

[4] De Giovanni R (1961). The effects of deuterium oxide on bacteria. Z Vererbungsl.

[5] Unno K, Kishido T, Morioka M, Okada S, Oku N (2003). Increased expression of Hsp70 for resistance to deuterium oxide in a yeast mutant cell line. Biological and Pharmaceutical Bulletin.

[6] Newo A, Pshenichnikova A, Skladnev D, Shvets V (2004). Deuterium oxide as a stress factor for the methylotrophic bacterium Methylophilus sp. B-7741. Microbiology.

[7] Meilleur F, Weiss KL, Myles DA (2009). Deuterium labeling for neutron structure-function-dynamics analysis. Methods Mol Biol.

[8] Paliy O, Bloor D, Brockwell D, Gilbert P, Barber J (2003). Improved methods of cultivation and production of deuteriated proteins from E. coli strains grown on fully deuteriated minimal medium. J Appl Microbiol.

[9] Smith KM, Liao JC (2011). An evolutionary strategy for isobutanol production strain development in Escherichia coli. Metabolic engineering.

[10] Blattner FR (1997). The complete genome sequence of *Escherichia coli* K-12. Science.

[11] Badran AH, Liu DR (2015). Development of potent *in vivo* mutagenesis plasmids with broad mutational spectra. Nature commun.

[12] Warren DJ (2011). Preparation of highly efficient electrocompetent Escherichia coli using glycerol/mannitol density step centrifugation. Anal Biochem.

[13] Kelpsas V (2019). Perdeuteration, large crystal growth and neutron data collection of *Leishmania mexicana* triose-phosphate isomerase E65Q variant. Acta Crystallogr F Struct Biol Commun.

[14] Scholz J, Besir H, Strasser C, Suppmann S (2013). A new method to customize protein expression vectors for fast, efficient and background free parallel cloning. BMC Biotechnol.

[15] Engman J, von Wachenfeldt C (2015). Regulated protein aggregation: a mechanism to control the activity of the ClpXP adaptor protein YjbH. Mol Microbiol.

[16] Schaaper RM (1993). Base selection, proofreading, and mismatch repair during DNA replication in Escherichia coli. J Biol Chem.

[17] Garen A, Levinthal C (1960). A fine-structure genetic and chemical study of the enzyme alkaline phosphatase of *E. coli* I. Purification and characterization of alkaline phosphatase. Biochim Biophys Acta.

[18] Goodall, E. C. A. *et al*. The essential genome of *Escherichia coli* K-12. *MBio* **9**, 10.1128/mBio.02096-17 (2018). 10.1128/mBio.02096-17 PMC5821084 29463657

[19] Serre L (2001). How methionyl-tRNA synthetase creates its amino acid recognition pocket upon L-methionine binding. J Mol Biol.

[20] Schmitt E, Meinnel T, Blanquet S, Mechulam Y (1994). Methionyl-tRNA synthetase needs an intact and mobile 332KMSKS336 motif in catalysis of methionyl adenylate formation. J Mol Biol.

[21] Lee K (2003). RraA: a protein inhibitor of RNase E activity that globally modulates RNA abundance in *E. coli*. Cell.

[22] Studier FW, Moffatt BA (1986). Use of bacteriophage T7 RNA polymerase to direct selective high-level expression of cloned genes. J Mol Biol.

