# Supplementary material for: Strain improvement of Escherichia coli K-12 for recombinant production of deuterated proteins
Source: Sci Rep. 2019 Nov 27;9:17694. doi: 10.1038/s41598-019-54196-w (PMC6881287; doi:10.1038/s41598-019-54196-w)
Supplement: Supplementary file 1 — Supplementary information [file 41598_2019_54196_MOESM1_ESM.pdf]

# Supplementary information

Strain improvement of *Escherichia coli* K-12 for recombinant production of deuterated proteins

Vinardas Kelpšas and Claes von Wachenfeldt

**Supplementary Table 1. Mutations identified by resequencing of *E. coli* MG1655.**

| Position[a] | Variant[b] | Reference base(s) | Variant base(s) | Annotations | Coding region change[c] | Protein change[d] |
|---|---|---|---|---|---|---|
| 257908 | SNV | G | A | Mobile element | 71+9G>A | |
| 2173361 | Deletion | CC | - | *gatC* | 915_916delGG | Val306fs |
| 3005223 | SNV | A | T | *ygeV* | 564T>A | |
| 3560456 | Insertion | - | G | *glpR* | 150_150+1insC | |
| 4296268 | SNV | T | C | Repeat region: RIP321 | 915_916delGG | |
| 4296381 | Insertion | - | CG | Repeat region: RIP321 | 564T>A | |

[a]Nucleotide position in the reference sequence Genbank entry: U00096.3
[b]Single nucleotide variant (SNV)
[c]Deletion (del), insertion (ins)
[d]Frame shift (fs)

**Supplementary Table 2. Mutations identified in *E. coli* Mut25[a].**

| Position[b] | Variant[c] | Reference base(s) | Variant base(s) | Annotations | Coding region change[d] | Protein change[e] |
|---|---|---|---|---|---|---|
| 7504 | SNV | G | T | *yaaJ* | 456C>A | |
| 83057 | SNV | G | A | *leuA* | 473C>T | Ala158Val |
| 107261 | SNV | T | C | *lpxC* | 705T>C | |
| 442544 | SNV | C | T | *thiI* | 996C>T | |
| 1236309 | SNV | A | G | *ycgB* | 933T>C | |
| 1818429 | SNV | A | G | *chbF* | 72T>C | |
| 1868484 | SNV | A | C | *yeaG* | 1577A>C | Gln526Pro |
| 2240328 | SNV | G | A | *mglB* | 21C>T | |
| 2281454 | SNV | G | A | *yejH* | 823G>A | Glu275Lys |
| 2405300 | SNV | T | C | | | |
| 2456410 | SNV | T | A | *sixA* | 403A>T | Ser135Cys |
| 2512406 | SNV | C | T | *mntH* | 301G>A | Ala101Thr |
| 2605605 | SNV | C | A | *hyfD* | 795C>A | |
| 2625620 | SNV | G | T | *ppx* | 506G>T | Cys169Phe |
| 2807480 | SNV | T | C | *proX* | 349T>C | Tyr117His |
| 2923174 | SNV | C | T | *truC* | 611G>A | Ser204Asn |
| 2952150 | SNV | G | A | *recD* | 312C>T | |
| 3006699 | SNV | G | A | *ygeW* | 438G>A | |
| 3017794 | SNV | T | A | *ygfK* | 1735T>A | Phe579Ile |
| 3103559 | SNV | G | A | *mutY* | 547G>A | Ala183Thr |
| 3254544 | SNV | C | T | *yhaK* | 226C>T | Arg76Cys |
| 3354872 | SNV | A | G | *gltB* | 148A>G | Lys50Glu |
| 3410247 | SNV | C | T | | | |
| 3543733 | SNV | G | A | *yhgA* | 567G>A | Met189Ile |
| 3618277 | SNV | G | A | *nikE* | 502G>A | Val168Ile |
| 3795223 | SNV | T | C | *waaF* | 295T>C | Phe99Leu |
| 3834747 | SNV | T | C | *yicJ* | 1183A>G | Lys395Glu |
| 3870912 | SNV | G | A | *dgoT* | 819C>T | |
| 4143036 | SNV | T | C | *frwC* | 507T>C | |
| 4286521 | SNV | T | C | *acs* | 851A>G | Tyr284Cys |
| 4411089 | SNV | T | G | *yjfJ* | 537T>G | |
| 4602331 | Deletion | G | - | *yjjP* | 528delC | Thr177fs |

[a]In addition to the listed mutations, the *E. coli* Mut25 strain had the same changes relative to the reference genome sequence of *E. coli* MG1655 (Genbank entry: U00096.3) as was identified in the used laboratory stock of MG1655 (Supplementary Table 1)

[b]Nucleotide position in the reference sequence Genbank entry: U00096.3

[c]Single nucleotide variant (SNV)

[d]Deletion (del)

[e]Frame shift (fs)

**Supplementary Figures**

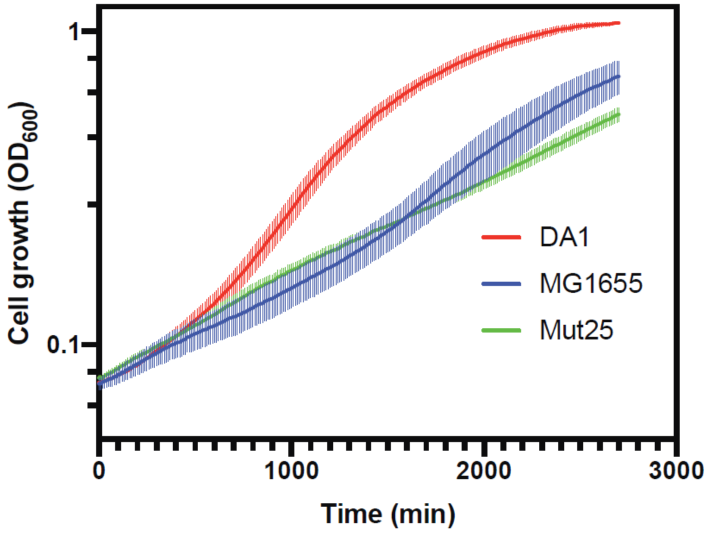


**Figure S1. Bioscreen C growth analysis of *E. coli* strains.**

*E. coli* strains MG1655, DA1 and Mut25 were grown in deuterated minimal medium supplemented with deuterated glycerol (DD-M9). Plates were incubated with continuous shaking at 37°C and $OD_{600}$ was measured every 10 min with 5 seconds of no shaking before each measurement. For each strain three biological replicates were analyzed. The mean and the standard error of the mean is plotted.

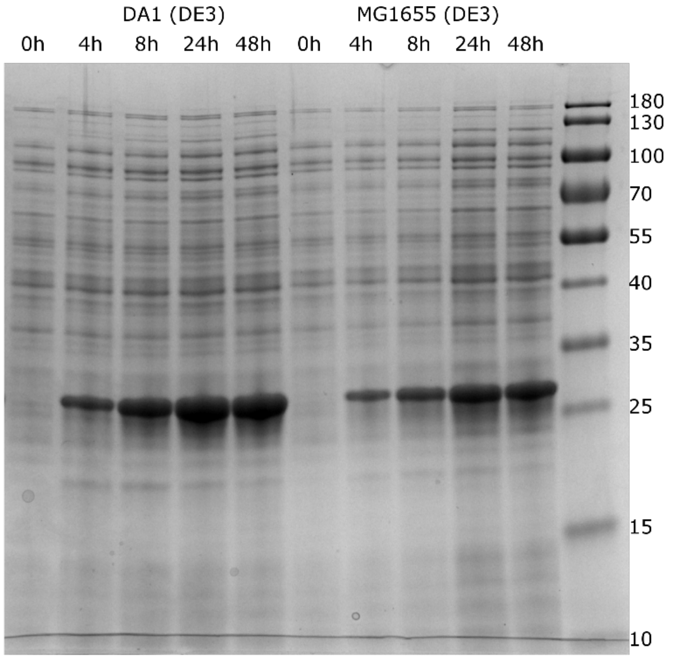


**Figure S2. Unedited version of Figure 4C.**

**Supplementary Figure 3.**

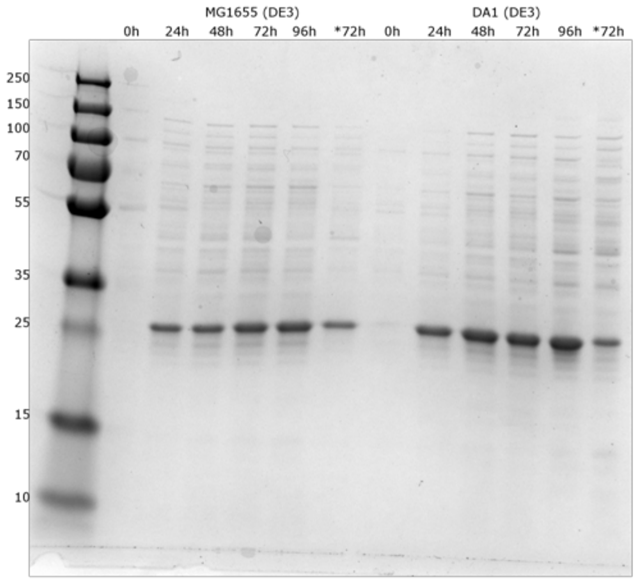


**Figure S3. Uncropped version of Figure 5.**
